# Clinical Significance of Prothrombin Time in Cholangiocarcinoma Patients with Surgeries

**DOI:** 10.1155/2019/3413969

**Published:** 2019-07-01

**Authors:** Hui-shan Wang, Xian-xiu Ge, Quan-peng Li, Jun-jie Nie, Lin Miao

**Affiliations:** ^1^Nanjing Medical University, 101 Longmian Avenue, Jiangning District, Nanjing 211166, Jiangsu Province, China; ^2^Medical Center for Digestive Diseases, The Second Affiliated Hospital of Nanjing Medical University, 121 Jiangjiayuan, Nanjing 210011, Jiangsu Province, China

## Abstract

**Background:**

Prothrombin time (PT) can predict survival in several types of malignancies. This study aims to investigate the predictive values of PT levels in patients with cholangiocarcinoma (CCA).

**Methods:**

We retrospectively analyzed the PT from 86 CCA patients who underwent curative resection in our hospital from December 2008 to August 2017. The relationship between PT and survival times was analyzed through univariate and multivariate analyses (Cox proportional hazards model). Kaplan–Meier curves and log-rank test were used to assess the effects of PT on overall survival (OS) and tumor recurrence-free survival (RFS).

**Results:**

Increased PT level was an effective predictor for OS (P = 0.021; hazard ratio (HR), 1.799) and RFS (P = 0.016; HR, 1.871) in CCA patients, independent of age, tumor differentiation, and TNM stage. In the low PT level group (PT < 12.3 s), patients showed a higher mean OS (23.03 m vs. 14.38 m, P = 0.0250) and RFS (17.78 m vs. 8.30 m, P = 0.0511) than those with high PT levels (PT ≥ 12.3 s). A highly significant association was observed between high PT level and shortened OS (P = 0.0373) and worse RFS (P = 0.0151).

**Conclusion:**

Preoperative increase in PT can serve as a simple but effective predictor of poor survival in CCA patients who undergo curative surgeries.

## 1. Introduction

Cholangiocarcinoma (CCA) is a malignancy arising from the intra or extrahepatic biliary epithelium, and it is characterized with its late diagnosis and fatal outcome. CCA is the second most common primary liver tumor, and it accounts for 10%–15% of all hepatobiliary malignancies. Hilar CCA constitutes 60%–70% of CCA, and distal and intrahepatic CCAs account for 20%–30% and 5%–10%, respectively. The pathogenesis of CCA is closely related to biliary inflammation, cholestasis, and liver inflammation, and its incidence is recently increasing worldwide, especially in western countries [1]. Surgery is still the only potentially curative treatment option for CCA but the prognosis of CCA patients is poor, with a five-year overall rate less than 35% [2]. In the past years, scientific research has focused on investigating the following markers for predicting outcomes of CCA: (i) tumor biomarkers, such as CA199 [3] and CEA [4]; (ii) inflammation-based prognostic factors, such as the C-reactive protein (CRP) and albumin (the combination of CRP and albumin, also known as modified Glasgow Prognostic Score, mGPS) [5]; (iii) neutrophil-lymphocyte ratio (NLR) [6, 7] and platelet-lymphocyte ratio (PLR) [8]; (iv) prognostic nutritional index [9]; (v) liver functions, such as ALP, T-BIL, D-BIL, and GGT [10]; and (vi) coagulation function, such as D-dimer (DD) [11]; which all provide useful information for predicting prognosis in patients with CCA. Increasing evidence suggests that coagulation activation, tumor progression, metastatic spread, and prognosis are closely related. For example, preoperative hyperfibrinogenemia is associated with tumor progression, inflammatory mediator, and poor OS in patients with gastric [12] and gallbladder cancers [13]. Moreover, hyperfibrinogenemia can also predict the response to trastuzumab in breast cancer [14]. And for pancreatic cancer patients, prothrombin time (PT) displays higher levels of prothrombin time (PT) than those of healthy people [15]. And it has been reported that postoperative PT-international normalized ratio (INR) can accurately predict the postoperative mortality of CCA patients after major hepatectomy with extrahepatic bile duct resection [16]. In addition, posthepatectomy liver failure in perihilar CCA patients is significantly associated with preoperative PT (PT INR) > 1.20 (OR = 3.22, P < 0.05) [17]. However, data about the role of preoperative PT levels in outcomes in CCA patients are limited. In the present study, we aim to explore whether PT levels before surgery can predict OS and tumor recurrence in CCA patients.

## 2. Methods

### 2.1. Patient Selection

The study cohort comprised 86 consecutive patients with CCA who underwent curative resection in the Second Affiliated Hospital of Nanjing Medical University from December 2008 to August 2017. These cases included 28 patients with distal CCA, 35 patients with hilar CCA and 23 patients with intrahepatic CCA. The inclusion criteria were as follows: (i) pathological diagnosis of CCA; (ii) underwent curative resection and no definite chemotherapy or radiotherapy was administered before surgery; and (iii) with coagulation function examination and blood routine test before surgery. Patients were excluded from this study if any of the following was present: (i) known congenital coagulation abnormality; (ii) continuous anticoagulant therapy (such as aspirin and clopidogrel) before the surgery; and (iii) arterial thromboembolism within the preceding 3 months and stroke or thrombosis within the preceding 6 months. The study was approved by the Ethics Committee of the Second Affiliated Hospital of Nanjing Medical University (Nanjing, China), and written informed consent was obtained from all participants. All aspects of the study related to human participants were in accordance with the ethical standards of the institutional and national research committee and the Helsinki Declaration.

### 2.2. Data Collection

All experimental data, including activated partial thromboplastin time (APTT), PT, INR, fibrinogen (FIB), fibrinogen degradation product (FDP), thrombin time (TT), D-dimer (DD), platelet count (PLT), platelet distribution width (PDW), mean platelet volume (MPV), lymphocyte count (LYM), neutrophil count (NEU), albumin (ALB), C-reactive protein (CRP), CA199, CA125, and CEA were collected at the time of admission, without any medical operation, like ERCP (Endoscopic Retrograde Cholangiopancreatography) or use of anticoagulants. The blood routine test was analyzed by a blood cell analyzer (Sysmex XN-1000), blood coagulation was tested by a completely automatic instrument (Sysmex CS5100), serum CRP concentration was measured by a nephelometric immunoassay (SIEMENS BNII), liver function was tested using automatic biochemical immunoassay analyzer (cobas 8000, Roche), and tumor biomarkers were tested by (cobas e 602, Roche). The laboratory calibration references for the preceding parameters were as follows: APTT, 24.0–39.0 s; PT, 11.0–13.0 s; INR, 0.80–1.20; FIB, 2.00–4.00 g/l; FDP, < 5 *μ*g/ml; TT, 14.0–21.0 s; DD, 0–1.0 *μ*g/ml; PLT, 100–300*∗*10^9^/L; PDW, 9-17 fL; MPV, 6-11.5 fL, LYM, 0.8-4.0*∗*10^9^/L; NEU, 2.0-7.5*∗*10^9^/L; ALB, 35-50 g/L; CRP, 0-10 mg/L; CA199, 0-37 U/ml; CA125, 0-35 U/ml; CEA, 0-10 ug/l. The Glasgow Prognostic Score (GPS) was allocated as previously described, combining preoperative serum CRP and albumin levels. Patients with elevated CRP level (>10 mg/l) and hypoalbuminemia (<35 g/l) were scored as 2, patients with only one of the two biochemical abnormalities were scored as 1, and patients with neither of the two abnormalities were scored as 0. The relationship between the preoperative values of these data and the tumor characteristics, including TNM stage, lymph node metastasis, nerve invasion, and differentiation, was evaluated. Tumor differentiation was determined according to the British Society of Gastroenterology guidelines on the management of CCA. Tumor staging was confirmed on the basis of the eighth edition of the American Joint Committee on Cancer TNM Staging Manual (AJCC, 8th ed., 2017).

### 2.3. Follow-Up Strategy

All patients were followed up by interview, telephone call, and network communication. Ultrasonography, computed tomography (CT) or magnetic resonance imaging scanning and tumor biomarkers, such as CA199 and CEA, were used to detect tumor recurrence, and the incidence of metastasis. RFS was defined as the interval between the date of surgery and the first recurrence or metastasis or from the date of surgery to the date of last follow-up patients without recurrence. OS was defined as the interval between surgery and death or the interval between surgery and the last observation for surviving patients. Data were censored at the last follow-up for living patients.

### 2.4. Statistical Analysis

All statistical analyses were performed using STATA software version 13 (Stata Corp LP, College Station, TX, USA). Data were shown as mean ± SD, median (min-max), case number, or % wherever applicable. RFS and OS were estimated with Kaplan–Meier analysis and compared using the log-rank test. Cox proportional hazards regression was carried out to identify the independent factors that can significantly affect patient prognosis. One-way ANOVA was used if the variance was uniform, and the rank sum test was adopted if the variance was nonuniform. All statistical tests were two-sided, and P< 0.05 was considered significant.

## 3. Results

### 3.1. Patient Characteristics

The mean age of the patients at the time of diagnosis was 62.3 years (range of 35–96 years), and the cohort included 48 men (55.8%) and 38 women (44.2%). The patients all received curative surgeries, and the R0 rate was 77.90% (67/86). The pathological features showed 82 cases of adenocarcinoma, 2 cases of signet-ring cell carcinoma, 1 case of sarcoma, and 1 case of squamous cell carcinoma. The average follow-up time after surgery was 20.82 months (range of 2.8–95.8 months). The demographics and clinical characteristics of patients with CCA in this study are presented in Table 1. FDP was excluded in our study because it was not inspected before surgery in 12 patients. Given that INR resulted from PT, it was also excluded during the further analysis.

### 3.2. Independent Prognostic Values of Preoperative PT Levels in Patients with CCA

A total of 75% of the indices were selected for a cut-off value to divide the patients into two groups, except that the age group was divided by the median age of 64, the ALB group was divided by 35 g/L, the CRP group was divided by 10 mg/L, and the CA199 group was divided by 37 U/ml. We separated the patients into two groups according to PT levels based on the cut-off value: low (<12.3 s) or high (≥12.3 s). Univariate and multivariate analyses were conducted to assess the predictors of OS (Table 2) and RFS (Table 3). The hazard ratios (HR) and 95% confidence intervals (CI) estimated from Cox regression models indicated that increased PT levels were strongly associated with OS (P = 0.041; HR, 1.670; 95% CI, 1.020–2.733) and RFS (P = 0.019; HR, 1.827; 95% CI, 1.105–3.018). Additionally, patients over 64 years old or those with high NLR (NLR> 6.17) or low ALB (ALB< 35 g/L) or poor tumor differentiation or high TNM stage may present a high possibility of poor RFS (P = 0.023, P=0.069, P=0.011, P < 0.001, and P = 0.003, respectively) and low OS (P = 0.017, P=0.020, P=0.032, P <0.001, and P = 0.015, respectively). After multivariate analysis, increased PT levels remained a highly significant predictor of OS (P = 0.021; HR, 1.799; 95% CI, 1.091–2.965) and RFS (P = 0.016; HR, 1.871; 95% CI, 1.121–3.123), independent of age, tumor differentiation, and TNM stage (Tables 2 and 3). Nevertheless, other coagulation function indices exerted no predictive effect on OS or RFS (all P > 0.05).

### 3.3. Relationships between Preoperative PT Level and CCA Survival

Patients with low PT levels showed a higher median OS (17.5 m ± 2.756 m vs. 8.0 m ± 2.919 m; 95% CI, 12–22.5 m vs. 5.0–16.0 m) and RFS (9.0 m ± 2.12 m vs. 6.0 m ± 1.576 m; 95% CI, 6.0–15. 0m vs. 2.5–8.0 m) than those with high PT levels. Furthermore, in the low PT level group, patients presented a higher mean OS (23.03 m vs. 14.38 m, P = 0.0250) with a significant statistical difference and higher mean RFS (17.78 vs. 8.30, P = 0.0511) with no statistical difference than those of patients in the high PT level group (Table 4).

**Figure 1:**
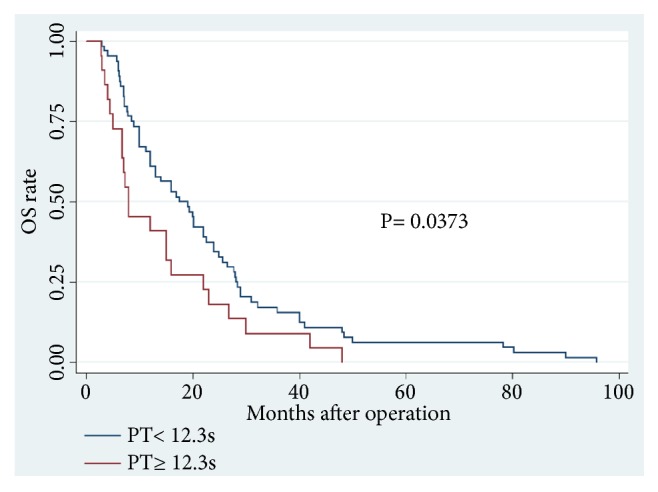
Overall survival in patients with cholangiocarcinoma according to PT levels (P=0.0373) (PT, prothrombin time; OS, overall survival).

**Figure 2:**
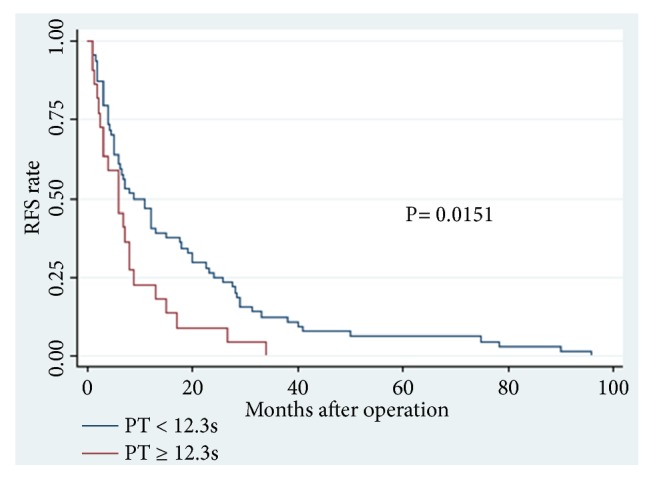
Recurrence-free survival in patients with cholangiocarcinoma according to PT levels (P=0.0151) (PT, prothrombin time; RFS, recurrence-free survival).

**Figure 3:**
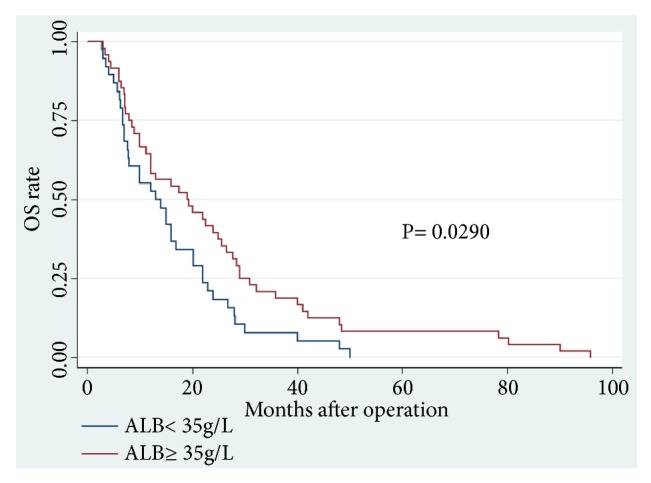
Overall survival in patients with cholangiocarcinoma according to ALB levels (P=0.0290) (ALB, albumin; OS, overall survival).

**Figure 4:**
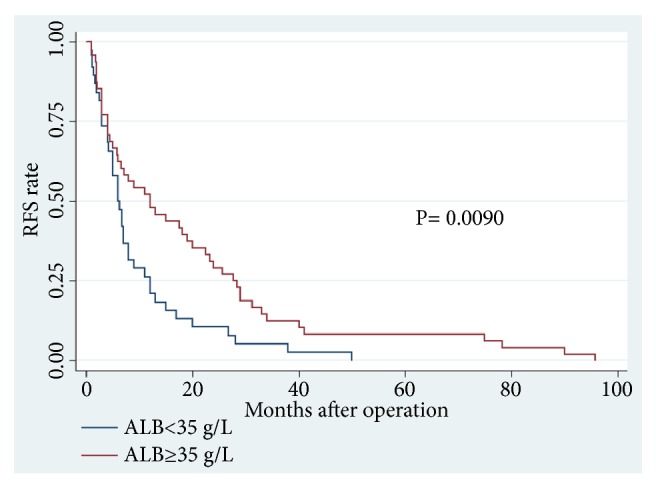
Recurrence-free survival in patients with cholangiocarcinoma according to ALB levels (P=0.0090) (ALB, albumin; RFS, recurrence-free survival).

**Figure 5:**
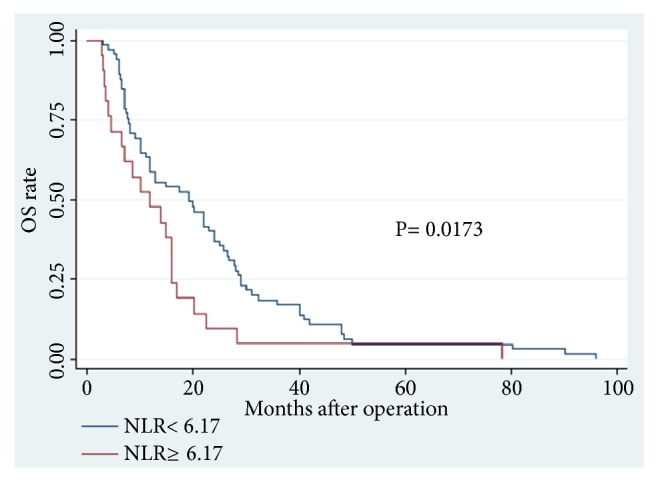
Overall survival in patients with cholangiocarcinoma according to NLR levels (P=0.0173) (NLR, neutrophil-lymphocyte ratio; OS, overall survival).

Among the 86 CCA patients, death occurred in 39 of 64 (61%) patients with low PT level and in 18 of 22 (82%) patients with high PT level (P = 0.074). Kaplan–Meier survival analysis demonstrated a highly significant association between high PT level and shortened OS (P = 0.0373, Kaplan–Meier method) (Table 5; Figure 1). Patients with high PT levels showed a significant trend toward poor RFS compared with that of patients with low PT levels (P = 0.0151, Kaplan–Meier method) (Table 5, Figure 2). In addition, patients with high ALB (>35 g/L) levels tend to have better OS (P = 0.0290, Kaplan–Meier method) (Table 5; Figure 3) and less opportunity to recurrence (P = 0.0090, Kaplan–Meier method) (Table 5; Figure 4). What is more, high NLR (>6.17) patients seem to have poor OS (P = 0.0173, Kaplan–Meier method) (Table 5; Figure 5) but not RFS (P = 0.0621, Kaplan–Meier method) (Table 5). However, no significant relation was observed between the other indices and OS or RFS (all P > 0.05); the Kaplan–Meier curves were not shown in the article due to the limit space.

## 4. Discussion

The CCA prognostic factors play an important role not only in advertising individuals, selecting treatments, and understanding the disease in the clinical field, but also in providing additional directions for further research. In the past decades, several laboratory abnormalities, including increased tumor biomarkers (e.g., CA199 [3] and CEA [4]), inflammation-based prognostic factors (e.g., mGPS [5], NLR [6, 7], PLR [8], and liver enzymes [10]), have been reported to be associated with poor prognosis in CCA patients. We included these parameters in our study and the results showed that the markers were not correlated to overall outcome, except ALB and NLR. Preoperative serum ALB reflects nutritional status; it decreases in response to systemic inflammation during malignant tumor growth, leading to hypoalbuminemia and weight loss, which may in turn affect the immune system of the cancer patients. Hypoalbuminemia therefore suggests systemic inflammation and immune suppression, in association with the initiation and progression of malignant tumors [5]. And NLR may be an important indicator of the inflammatory state of patient at the time of surgery. These evidences may explain the correlation of ALB and NLR with prognosis of CCA patients observed in this study. Furthermore, we specially explored the relationship between coagulation parameters, including PLT, PDW, MPV, PLR, APTT, PT, INR, FIB, TT, DD, and CCA patient survival (OS and RFS). And results showed that CCA patients with increased preoperative PT levels exerted poorer OS and RFS. After multivariate analysis, increased preoperative PT level remained as statistically significant predictors of poor outcomes, which may mean elevated PT levels in patients with poor outcome directly reflects pathophysiological aspects of the disease, but not just an epiphenomenon.

Approximately 94% of cancer patients suffer from one or more abnormal coagulation functions. This situation is severe in patients with advanced tumors with systemic multiple metastases [18]. Several cases [19, 20] reported that patients with CCA was complicated by Trousseau's syndrome (malignancy-related thromboembolism), which is commonly attributed to a cancer-related hypercoagulable state, chronic disseminated intravascular coagulopathy, or tumor embolism. On the basis of high blood coagulation and hyperfibrinolysis, endothelial cells promote disease progression to advanced stages and multiple metastases. Chong De Lu et al. [21] showed that disease progression in patients with intrahepatic CCA and portal vein tumor thrombus (PVTT) is more advanced than those without PVTT; that is, patients with PVTT were more likely to present regional lymph node metastasis. Additionally, PVTT is an independent prognostic factor for poor OS and disease-free survival.

PT is an index reflecting the extrinsic pathway of coagulation, which is used to determine the clotting tendency of blood in the measurement of warfarin dosage, liver damage and vitamin K status. Furthermore, PT is routinely and easily measured in laboratory test before surgery, and it can be inspected in most hospitals. Therefore, PT may serve as a useful and cost-effective predictor of outcome in clinical practice. Both clinical and experimental evidence support the idea that the coagulation and fibrinolysis activation may play an important role in tumor survival. Qi Y et al. [22] found that the PT level in NSCLC patients was significantly higher than that in the healthy persons (P<0.05). Moreover, the survival rate of NSCLC patients with prolonged PT (PT > 12.1 s) is significantly reduced (P = 0.02, HR, 1.712). Similarly, Schweitzer N et al. [23] reported that prolonged PT (>75%) is significantly related to poor survival in patients with intrahepatic CCA after surgery (p < 0.001, HR, 4.20). In our study, a significant association existed between the increased PT level and shortened OS (P = 0.021; HR, 1.799), which was consistent with previous results. We also found that increased PT level is a predictor of tumor recurrence survival (P = 0.016; HR, 1.871). However, a few limitations exist in this study. First, the sample size of our study is small because only approximately one-third of the patients with CCA are available for curative treatment [1]. A large sample may have presented largely valid findings. Second, given that the coagulation function can be affected with other comorbidities, the exclusion criteria may not be versatile. Detailed data on the clinical variables of each patient regarding smoking and drinking are also necessary. Third, we failed to further analyze the data about site recurrence due to a few missing values.

In conclusion, this study characterizes the potential of PT level in predicting the outcome of patients with CCA. Preoperative increase in PT can serve as simple but effective predictor of poor survival in patients with CCA who undergo curative surgeries.

## Figures and Tables

**Table 1:** Demographics and clinical characteristics of patients with cholangiocarcinoma.

Variable	Mean ± SD (Frequency)	Range
Gender (M/F)	48/38	-
Age	62.32±11.17	35-96
PLT, 10^9^/L	208.81±72.77	48.6-414.0
PDW, fL	17.17±8.58	10.6-53.3
MPV, fL	11.70±1.71	6.6-19.1
LYM, 10^9^/L	1.24±0.50	0.28-3.01
NEU, 10^9^/L	5.63±2.68	1.1-14.04
PLR	189.61±106.14	58.93-883.93
NLR	5.36±3.84	1-19.65
APTT,s	28.50±6.31	19.0-59.8
PT,s	11.53±1.48	9.3±17.9
INR	0.996±0.124	0.8-1.52
FIB, g/L	3.94±1.32	1.2-7.9
TT, s	18.35±2.42	15.2-34.1
DD, ug/mL	1.14±1.79	0.08-12.96
ALB, g/L	36.17±6.15	20-56
CRP, mg/L	38±47.18	1-240
GPS (0/1-2)	19/67	-
CA199, U/ml	1108.09±6780.71	0.6-59575
CA125, U/ml	36.50±47.98	3.5- 246.1
CEA, ng/ml	4.97±6.78	0.2-44.83
Differentiation (Middle-High/Low)	56/29	-
Lymph (+)/(-)	39/47	-
Nerve (+)/(-)	60/26	-
Stage (I-II/III-IVA)	62/24	-
Recurrence (Y/N)	65/21	-
Die (Y/N)	57/29	-
OS, months	20.82±18.97	2.8-95.8
RFS, months	15.35±19.14	1.0-95.8

PLT, platelet count, PDW, platelet distribution width, MPV, mean platelet volume, LYM, lymphocyte count NEU, neutrophil count, PLR, platelet-lymphocyte ratio, NLR, neutrophil-lymphocyte ratio, ALB, albumin, CRP, C-reactive protein, GPS, Glasgow Prognostic Score, APTT, activated partial thromboplastin time, PT, prothrombin time, INR, international normalized ratio, FIB, fibrinogen, TT, thrombin time, DD: D-dimer, Lymph: lymph node metastasis, and Nerve, nerve invasion. OS, overall survival and RFS, recurrence-free survival.

**Table 2:** Univariate and multivariate analyses of relationship between various clinical factors and overall survival (OS).

Variable	Category	Obs	Univariate analysis; OS			Multivariate analysis; OS		
			HR	P	95%CI	HR	P	95%CI
Gender	M/F	48/38	0.747	0.194	0.481-1.160			
Age	<64/≥64	40/46	*1.709*	*0.017*	*1.102-2.652*	1.478	0.097	0.932-2.344
PLT, 10^9^/L	<258.67/≥258.67	64/22	0.652	0.090	0.397-1.069			
PDW, fL	<17.99/>=17.99	64/22	0.916	0.726	0.560-1.497			
MPV, fL	<12.46/>=12.46	64/22	1.24	0.388	0.761- 2.021			
LYM, 10^9^/L	<1.5/>=1.5	64/22	0.636	0.075	0.387-1.046			
NEU, 10^9^/L	<7.12/>=7.12	64/22	1.160	0.553	0.711-1.891			
PLR	<240.18/>=240.18	66/20	0.940	0.814	0.560-1.577			
NLR	<6.17/>=6.17	65/21	*1.820*	*0.020*	*1.098-3.018*	1.457	0.178	0.843-2.517
APTT, s	<30.8/≥30.8	64/22	0.868	0.577	0.529-1.426			
PT, s	<12.3/≥12.3	64/22	*1.670*	*0.041*	*1.020-2.733*	*1.829*	*0.036*	*1.042-3.212*
INR	<1.07/≥1.07	64/22	*1.670*	*0.041*	*1.020-2.733*			
FIB, g/l	<4.49/≥4.49	64/22	1.393	0.196	0.843-2.304			
TT, s	<18.75/≥18.75	65/21	0.853	0.528	0.519-1.399			
DD, ug/ml	<1.11/≥1.11	63/21	1.263	0.341	0.781-2.043			
ALB, g/L	<35/≥35	38/48	*0.618*	*0.032*	*0.397-0.960*	1.238	0.459	0.704-2.176
CRP, mg/L	<10/≥10	25/61	1.304	0.272	0.812-2.092			
GPS	0/1-2	19/67	1.198	0.494	0.714-2.011			
CA199, U/ml	<37/>=37	28/58	1.295	0.269	0.818-2.050			
CA125, U/ml	<36.49/≥36.49	59/27	0.970	0.896	0.611-1.537			
CEA, ng/ml	<5.1/≥5.1	57/29	1.24	0.350	0.79-1.96			
Differentiation	Middle-High/Low	57/29	*3.382*	*0.001*	*2.033-5.628*	*3.046*	*0.001 *	*1.667-5.566*
Lymph	(+)/(-)	39/47	0.712	0.127	0.459-1.102			
Nerve	(+)/(-)	60/26	0.756	0.240	0.475-1.205			
Stage	I-II/III-IVA	62/24	*1.840*	*0.015*	*1.126-3.008*	1.621	0.062	0.975-2.695

Obs, observation, PLT, platelet count, PDW, platelet distribution width, MPV, mean platelet volume, LYM, lymphocyte count NEU, neutrophil count, PLR, platelet-lymphocyte ratio, NLR, neutrophil-lymphocyte ratio, ALB, albumin, CRP, C-reactive protein, GPS, Glasgow Prognostic Score, APTT, activated partial thromboplastin time, PT, prothrombin time, INR, international normalized ratio, FIB, fibrinogen, TT, thrombin time, DD: D-dimer, Lymph: lymph node metastasis, and Nerve, nerve invasion. OS, overall survival and RFS, recurrence-free survival.

**Table 3:** Univariate and multivariate analyses of relationship between various clinical factors and recurrence-free survival (RFS).

Variable	Category	Obs	Univariate analysis; RFS			Multivariate analysis; RFS		
			HR	P	95%CI	HR	P	95%CI
Gender	M/F	48/38	0.786	0.278	0.508-1.215			
Age	<64/≥64	40/46	*1.650*	*0.023*	*1.071-2.542*	1.336	0.215	0.845-2.113
PLT, 10^9^/L	<258.67/≥258.67	64/22	0.677	0.124	0.413-1.112			
PDW, fL	<17.99/>=17.99	64/22	0.996	0.986	0.609-1.627			
MPV, fL	<12.46/>=12.46	64/22	1.101	0.700	0.675-1.794			
LYM, 10^9^/L	<1.5/>=1.5	64/22	0.691	0.143	0.422-1.133			
NEU, 10^9^/L	<7.12/>=7.12	64/22	1.147	0.582	0.704-1.868			
PLR	<240.18/>=240.18	66/20	0.981	0.941	0.586-1.642			
NLR	<6.17/>=6.17	65/21	1.590	0.069	0.965-2.622			
APTT, s	<30.8/≥30.8	64/22	0.950	0.838	0.578-1.559			
PT, s	<12.3/≥12.3	64/22	*1.827*	*0.019*	*1.105-3.018*	*1.826*	*0.033*	*1.049-3.177*
INR	<1.07/≥1.07	64/22	*1.827*	*0.019*	*1.105-3.018*			
FIB, g/l	<4.49/≥4.49	64/22	1.240	0.397	0.753-2.04			
TT, s	<18.75/≥18.75	65/21	0.800	0.379	0.487-1.315			
DD, ug/mL	<1.11/≥1.11	63/21	1.410	0.162	0.871-2.285			
ALB, g/L	<35/≥35	38/48	*0.561*	*0.011*	*0.359-0.877*	0.939	0.820	0.547-1.612
CRP, mg/L	<10/≥10	25/61	1.284	0.301	0.799-2.064			
GPS	0/1-2	19/67	1.289	0.338	0.767-2.167			
CA199, U/ml	<37/>=37	28/58	1.195	0.447	0.755-1.889			
CA125, U/ml	<36.49/≥36.49	59/27	0.932	0.765	0.589-1.476			
CEA, ng/ml	<5.1/≥5.1	57/29	1.120	0.626	0.711-1.763			
Differentiation	Middle-High/Low	57/29	*3.384*	*0.001*	*2.021-5.667*	*3.090*	*0.001 *	*1.748-5.461*
Lymph	(+)/(-)	39/47	0.690	0.094	0.447-1.064			
Nerve	(+)/(-)	60/26	0.692	0.124	0.433-1.107			
Stage	I-II/III-IVA	62/24	*2.154*	*0.003*	*1.295-3.583*	*2.040*	*0.008*	*1.201-3.467*

Obs, observation, PLT, platelet count, PDW, platelet distribution width, MPV, mean platelet volume, LYM, lymphocyte count, NEU, neutrophil count, PLR, platelet-lymphocyte ratio, NLR, neutrophil-lymphocyte ratio, ALB, albumin, CRP, C-reactive protein, GPS, Glasgow Prognostic Score, APTT, activated partial thromboplastin time, PT, prothrombin time, INR, international normalized ratio, FIB, fibrinogen, TT, thrombin time, DD: D-dimer, Lymph: lymph node metastasis, and Nerve, nerve invasion. OS, overall survival and RFS, recurrence-free survival.

**Table 4:** Analysis of overall survival (OS) and recurrence-free survival (RFS) in different groups.

Variable	Category	Mean OS	P value	Mean RFS	P value
Gender	Male/Female	18.60vs23.62	0.2993†	13.20vs18.08	0.3481†
Age	<64/≥64	25.68vs16.59	*0.0163 * **†**	19.69vs11.58	*0.0076*†
PLT, 10^9^/L	<258.67/≥258.67	18.91vs26.36	0.1127	13.97vs19.38	0.2554
PDW, fL	<17.99/>=17.99	20.13vs22.82	0.8045†	14.76vs17.08	0.6263
MPV, fL	<12.46/>=12.46	21.64vs18.41	0.4936	15.58vs14.70	0.8530
LYM, 10^9^/L	<1.5/>=1.5	19.12vs25.76	0.1575	14.27vs18.5	0.3745
NEU, 10^9^/L	<7.12/>=7.12	21.31vs19.37	0.6817	15.95vs13.61	0.6240
PLR	<240.18/>=240.18	20.50vs21.87	0.8620†	14.92vs16.79	0.7319†
NLR	<6.17/>=6.17	22.79vs14.7	0.0894	16.87vs10.65	0.1972
APTT, s	<30.8/≥30.8	20.36vs22.12	0.7114	15.25vs15.66	0.9304
PT, s	<12.3/≥12.3	23.03vs14.38	*0.0250 * **†**	17.78vs8.30	*0.0511*†
INR	<1.07/≥1.07	23.03vs14.38	*0.0250 * **†**	17.78vs8.30	*0.0511*†
FIB, g/L	<4.49/≥4.49	22.31vs16.48	0.7572†	16.74vs11.31	0.8829†
TT, s	<18.75/≥18.75	20.05vs23.19	0.5128	14.26vs18.75	0.3523
DD, ug/mL	<1.11/≥1.11	22.05vs17.63	0.3354	16.68vs11.93	0.3047
ALB, g/L	<35/≥35	15.99vs24.64	0.0725†	9.65vs19.87	*0.0426 * **†**
CRP, mg/L	<10/≥10	23.29vs19.80	*0.0495 * **†**	16.96vs14.69	*0.0235 * **†**
GPS	0/1-2	22.68vs20.29	0.0669†	17.12vs14.85	*0.0238 * **†**
CA199, U/ml	<37/>=37	24.07vs19.24	0.2715	17.33vs14.40	0.5086
CA125, U/ml	<36.49/≥36.49	20.60vs21.30	0.8750	15.09vs15.93	0.8515
CEA, ng/ml	<5.1/≥5.1	22.34vs17.82	0.6709†	16.27vs13.56	0.7181†
Differentiation	Middle-High/Low	25.94vs10.74	*0.0001*†	20.26vs 5.69	*0.0001*†
Lymph	(+)/(-)	17.41vs23.64	0.1302	11.57vs18.49	0.0955
Nerve	(+)/(-)	19.07vs24.85	0.1965	13.06vs20.64	0.0920
Stage	I-II/III-IVA	23.40vs14.15	*0.0430*†	18.41vs 7.45	*0.0199*†

†: rank sum test is used due to the uniform variance. The variance of other groups is uniform; therefore one-way ANOVA is used. PLT, platelet count, PDW, platelet distribution width, MPV, mean platelet volume, LYM, lymphocyte count NEU, neutrophil count, PLR, platelet-lymphocyte ratio, NLR, neutrophil-lymphocyte ratio, ALB, albumin, CRP, C-reactive protein, GPS, Glasgow Prognostic Score, APTT, activated partial thromboplastin time, PT, prothrombin time, INR, international normalized ratio, FIB, fibrinogen, TT, thrombin time, DD: D-dimer, Lymph: lymph node metastasis, and Nerve, nerve invasion. OS, overall survival and RFS, recurrence-free survival.

**Table 5:** Log-rank test for overall survival (OS) and recurrence-free survival (RFS).

Variable	Category	P value of log-rank test for OS	P value of log-rank test for RFS
Age	<64/≥64	*0.0146*	*0.0198*
PLT, 10^9^/L	<258.67/≥258.67	0.0846	0.1154
PDW	<17.99/>=17.99	0.7234	0.9862
MPV	<12.46/>=12.46	0.3825	0.6952
LYM	<1.5/>=1.5	0.0694	0.1348
NEU	<7.12/>=7.12	0.5483	0.5754
PLR	<240.18/>=240.18	0.8121	0.9405
NLR	<6.17/>=6.17	*0.0173*	0.0621
APTT, s	<30.8/≥30.8	0.5730	0.8356
PT, s	<12.3/≥12.3	*0.0373*	*0.0151*
INR	<1.07/≥1.07	*0.0373*	*0.0151*
FIB, g/l	<4.49/≥4.49	0.1900	0.3878
TT, s	<18.75/≥18.75	0.9689	0.7037
DD, ug/ml	<1.14≥1.14	0.3356	0.1537
ALB	<35/≥35	*0.0290*	*0.0090*
CRP	<10/≥10	0.2657	0.2919
GPS	0/1-2	0.4890	0.3282
CA199	<37/>=37	0.2634	0.4389
CA125	<36.49/≥36.49	0.8950	0.7607
CEA	<5.1/≥5.1	0.3447	0.6197
Differentiation	Middle-high/low	*0.0001*	*0.0001*
stage	I-II/III-IVA	*0.0126*	*0.0020*

Obs, observation, PLT, platelet count, PDW, platelet distribution width, MPV, mean platelet volume, LYM, lymphocyte count NEU, neutrophil count, PLR, platelet-lymphocyte ratio, NLR, neutrophil-lymphocyte ratio, ALB, albumin, CRP, C-reactive protein, GPS, Glasgow Prognostic Score, APTT, activated partial thromboplastin time, PT, prothrombin time, INR, international normalized ratio, FIB, fibrinogen, TT, thrombin time, and DD: D-dimer.

## Data Availability

The datasets analyzed during the current study and the statistic details are available from the corresponding author upon reasonable request.
